# Adoption and diffusion of SGLT2 inhibitors among cardiologists in medicare part D, 2019–2023: a provider-level observational study

**DOI:** 10.1186/s12872-026-06460-x

**Published:** 2026-08-13

**Authors:** Taemin Kim, Daniel Ryan, Jacob Joseph

**Affiliations:** 1 The Warren Alpert Medical School, Brown University, Providence, RI USA; 2 Cardiology Section, VA Providence Healthcare System, 830 Chalkstone Avenue, Providence, RI 02908 USA; 3 Massachusetts Veterans Epidemiology Research and Information Collaborative (MAVERIC), VA Boston Healthcare System, Boston, MA USA

**Keywords:** Sodium-glucose cotransporter-2 inhibitors, Heart failure, Medicare Part D, Cardiology, Prescribing diffusion, Geographic variation

## Abstract

**Background:**

Sodium–glucose cotransporter-2 (SGLT2) inhibitors are guideline-directed therapies for heart failure, yet real-world diffusion into cardiology practice remains incompletely characterized. This study evaluated adoption, prescribing intensity, subspecialty variation, geographic variation, and concentration of SGLT2 inhibitor use among cardiologists in Medicare Part D from 2019 to 2023.

**Methods:**

We conducted an observational, provider-level study using Medicare Part D Prescriber–Provider Public Use Files, including all clinicians with a primary specialty designation of cardiology. Primary analyses focused on empagliflozin and dapagliflozin. Outcomes included annual observable adoption (presence of a non-suppressed prescribing record), prescribing intensity among adopters, prescribing concentration (raw and normalized for overall Part D prescribing volume), adoption by cardiology subspecialty, total drug spending, and state-level variation with confidence intervals.

**Results:**

Among approximately 23,000 to 24,000 cardiologists annually, observable adoption increased from 4.5% in 2019 to 56.1% in 2023, with acceleration after 2021. Median prescribing intensity among adopters rose from 23 claims (interquartile range [IQR] 15–43) to 43 claims (IQR 21–90); early adopters prescribed nearly three times the intensity of late adopters after adjustment (incidence rate ratio, 2.96; 95% CI, 2.85–3.08). By 2023, adoption reached 91.6% among cardiologists with a subspecialty designation in advanced heart failure/transplant cardiology, compared with 56.4% among general cardiologists and 49.7% among interventional cardiologists (*P* < 0.001). Prescribing by raw claim volume remained concentrated, with the top 10% of prescribers accounting for 40.6% of total claims in 2023; this concentration was substantially attenuated, to 21.4%, after normalizing for each cardiologist’s overall Part D prescribing volume. Crude adoption varied substantially by state (25.6%-75.0% in 2023), though low-denominator states showed considerable estimate instability after empirical Bayes adjustment. Total Medicare Part D spending increased from $33.2 million in 2019 to $1.1 billion in 2023 in nominal dollars (28-fold in inflation-adjusted terms).

**Conclusions:**

Observable SGLT2 inhibitor adoption among cardiologists increased rapidly, but a substantial portion of apparent geographic heterogeneity and prescribing concentration reflects practice volume and subspecialty composition rather than adoption behavior alone. Adoption approached ceiling levels among advanced heart failure/transplant cardiologists, while general and interventional cardiologists lagged behind, identifying a specific target for future implementation efforts.

**Supplementary Information:**

The online version contains supplementary material available at https://doi.org/10.1186/s12872-026-06460-x.

## Background

Heart failure (HF) affects approximately 6.7 million adults in the United States and remains a leading cause of hospitalization and health care expenditure [1, 2]. Despite the availability of multiple disease-modifying therapies, outcomes remain suboptimal, and uptake of guideline-directed medical therapy (GDMT) has historically lagged behind clinical trial evidence [3].

SGLT2 inhibitors were initially developed as glucose-lowering agents for type 2 diabetes mellitus but subsequently demonstrated reductions in heart failure hospitalization and cardiovascular events independent of glycemic effects [4–7]. Subsequent dedicated heart failure trials established benefit across the LVEF spectrum, including HF with reduced, mildly reduced, and preserved ejection fraction [5–8]. This is particularly important for HF with preserved ejection fraction, a population with historically limited disease-modifying treatment options. Beyond heart failure, SGLT2 inhibitors have demonstrated additional cardiovascular benefits, further expanding their role in cardiovascular practice [4, 9, 10].

Contemporary clinical practice guidelines incorporate SGLT2 inhibitors as foundational HF therapy across the LVEF continuum [2, 11]. When added to angiotensin receptor-neprilysin inhibitors, β-blockers, and mineralocorticoid receptor antagonists, SGLT2 inhibitors are estimated to reduce the composite risk of cardiovascular death or heart failure hospitalization by approximately 60% compared with conventional therapy and may confer 3 to 8 additional years free from major HF events, depending on age. Despite these benefits, real-world uptake has been incomplete and heterogeneous. Clinical inertia persists, with current research finding prescriber unfamiliarity with cardiovascular context being a significant barrier; many cardiologists are hesitant to prescribe SGLT2 inhibitors to patients without diabetes due to their historical role as antihyperglycemic agents. Additional barriers include medication cost and formulary restrictions [12–14].

Although prior studies have described aggregate prescribing trends and implementation barriers, less is known about diffusion dynamics at the provider level, including timing of adoption, prescribing intensity among adopters, geographic variation, and the degree of concentration among early prescribers. Understanding these patterns is critical because uneven adoption may influence patient access, regional disparities, and health care spending. This study examines adoption and prescribing patterns of SGLT2 inhibitors among cardiologists in the Medicare Part D program from 2019 to 2023, spanning early diffusion through formal guideline incorporation. 

## Methods

### Study design and data source

We conducted an observational, provider-level study using the Medicare Part D Provider-and-Drug Public Use Files (PUFs) from 2019 to 2023. These PUFs provide annual prescribing volume and drug costs for Part D medications, along with prescriber characteristics including National Provider Identifier (NPI), specialty designation, and state. The unit of analysis was the clinician-year.

### Study population

For each study year, we identified clinicians with a primary specialty corresponding to cardiology, defined using case-insensitive string matching on the prescriber specialty field (terms containing “cardio”). The cardiologist denominator was constructed as a year-specific panel consisting of cardiologists observed in each year’s Part D file, such that clinicians were included only if they appeared in that year’s dataset.

### Exposure: SGLT2 inhibitors

In the primary analysis, SGLT2 inhibitor prescribing was defined using generic drug names for empagliflozin and dapagliflozin, identified using case-insensitive pattern matching of the generic name field; fixed-dose combination products (e.g., empagliflozin/metformin) were included in this match. In a sensitivity analysis, the exposure definition was expanded to include canagliflozin and ertugliflozin; this changed annual adoption estimates by ≤ 0.1% points in every study year, indicating the primary two-drug definition is not a meaningful source of bias.

### Outcomes

Primary outcomes were annual adoption, prescribing intensity among adopters, and prescribing concentration. Adoption was operationalized as the presence of a non-suppressed SGLT2 inhibitor prescribing record for the clinician-year in the Medicare Part D Prescriber PUF. Because CMS suppresses provider-drug rows with low claim counts, this measure should be interpreted as observable rather than complete adoption; a cardiologist with sufficiently low-volume SGLT2 inhibitor use could be misclassified as a non-adopter.

Prescribing intensity was measured as total annual SGLT2 inhibitor claims per adopting cardiologist, summarized using medians and interquartile ranges. Concentration was quantified as the share of total annual SGLT2 inhibitor claims attributable to high-volume prescribers, focusing on the top 1%, 5%, 10%, and 25% of adopters within each year. Secondary outcomes included total annual SGLT2 inhibitor drug costs and geographic variation in adoption across states.

### Construction of clinician-year measures and handling of CMS suppression

We cleaned numeric fields while preserving CMS-suppressed values as missing rather than recoding them as zero. Adoption was identified solely from the presence of an SGLT2 inhibitor row for the clinician-year. For adopter clinician-years, SGLT2 inhibitor utilization and drug costs were aggregated across included rows; totals were treated as missing if any contributing component was suppressed to avoid underestimation. For non-adopters, aggregated utilization and cost measures were set to zero. Derived per-claim and per-beneficiary measures were calculated only when numerators and denominators were non-suppressed and greater than zero. Drug costs were analyzed as reported in the Medicare Part D PUFs.

### Statistical analysis

We summarized annual cardiologist counts, adoption rates, and prescribing intensity from 2019 to 2023. Adoption was calculated as the proportion of cardiologists prescribing any SGLT2 inhibitor in each year. Intensity and spending summaries were restricted to adopter-years with non-suppressed values.

To evaluate concentration, we restricted analyses to adopter-years with observable claims and costs. Within each year, adopting cardiologists were ranked by total annual claims, and we calculated the proportion of total annual claims attributable to top prescribers; parallel calculations were performed for total annual drug costs. Suppression-related exclusions were quantified (Table S1).

To characterize geographic variation, we estimated state-level observable adoption rates with Wilson score 95% confidence intervals. To assess estimate stability, we additionally fit a random-intercept logistic model to obtain empirical Bayes–shrunk adoption estimates for each state. District of Columbia was omitted due to incomplete reporting. For mapping, non-state or non-mappable codes were excluded. Estimate stability by denominator size is addressed directly through Wilson confidence intervals and empirical Bayes shrinkage rather than a categorical low-denominator flag.

In supplemental analyses, cardiologists were classified as early adopters (2019–2021) or late adopters (2022–2023). Because annual SGLT2 inhibitor claims are a right-skewed count outcome, we fit negative binomial regression models (Poisson dispersion ratio = 87.5, confirming overdispersion) among adopter-years with non-suppressed claims, adjusting for calendar year and, in a secondary model, specialty category and state; standard errors were clustered by NPI to account for repeated observations per clinician.

### Subspecialty stratification

Cardiologists were further classified into three subspecialty categories based on the prescriber specialty field: general cardiology, interventional cardiology, and advanced heart failure/transplant cardiology. Adoption and prescribing intensity were compared across these groups using chi-square tests.

### Volume-normalized prescribing intensity

To evaluate whether prescribing concentration reflected adoption behavior or overall practice volume, we calculated each cardiologist’s total Part D claims across all drugs in the same year and expressed SGLT2 inhibitor claims as a rate per 1,000 total claims. Concentration was recalculated after ranking cardiologists by this normalized rate.

### Stable panel sensitivity analysis

As a sensitivity analysis, we restricted the cohort to cardiologists observed in all five study years to assess whether the dynamic annual denominator affected adoption trends.

### Software

All analyses were performed in R using tidyverse and data.table for data management, ggplot2 for visualization, and sf/tigris for geographic mapping. Figures and tables were generated programmatically from the analytic dataset derived from the Medicare Part D PUFs.

### Ethics

This study used publicly available, deidentified PUFs and did not include beneficiary-level identifiable information; it was therefore considered non-human subjects research or exempt, consistent with institutional policy.

### Artificial intelligence

No artificial intelligence tools were used in the conduct of this study or the preparation of this manuscript.

## Results

Across the study period, approximately 23,000 to 24,000 cardiologists were observed annually in the Medicare Part D prescriber files. Adoption of SGLT2 inhibitors increased rapidly between 2019 and 2023, accompanied by rising prescribing intensity, and increased Medicare spending, though diffusion remained geographically heterogeneous and prescribing persisted as highly concentrated among a small subset of providers.

### National trends in adoption, utilization, and spending

**Fig. 1:**
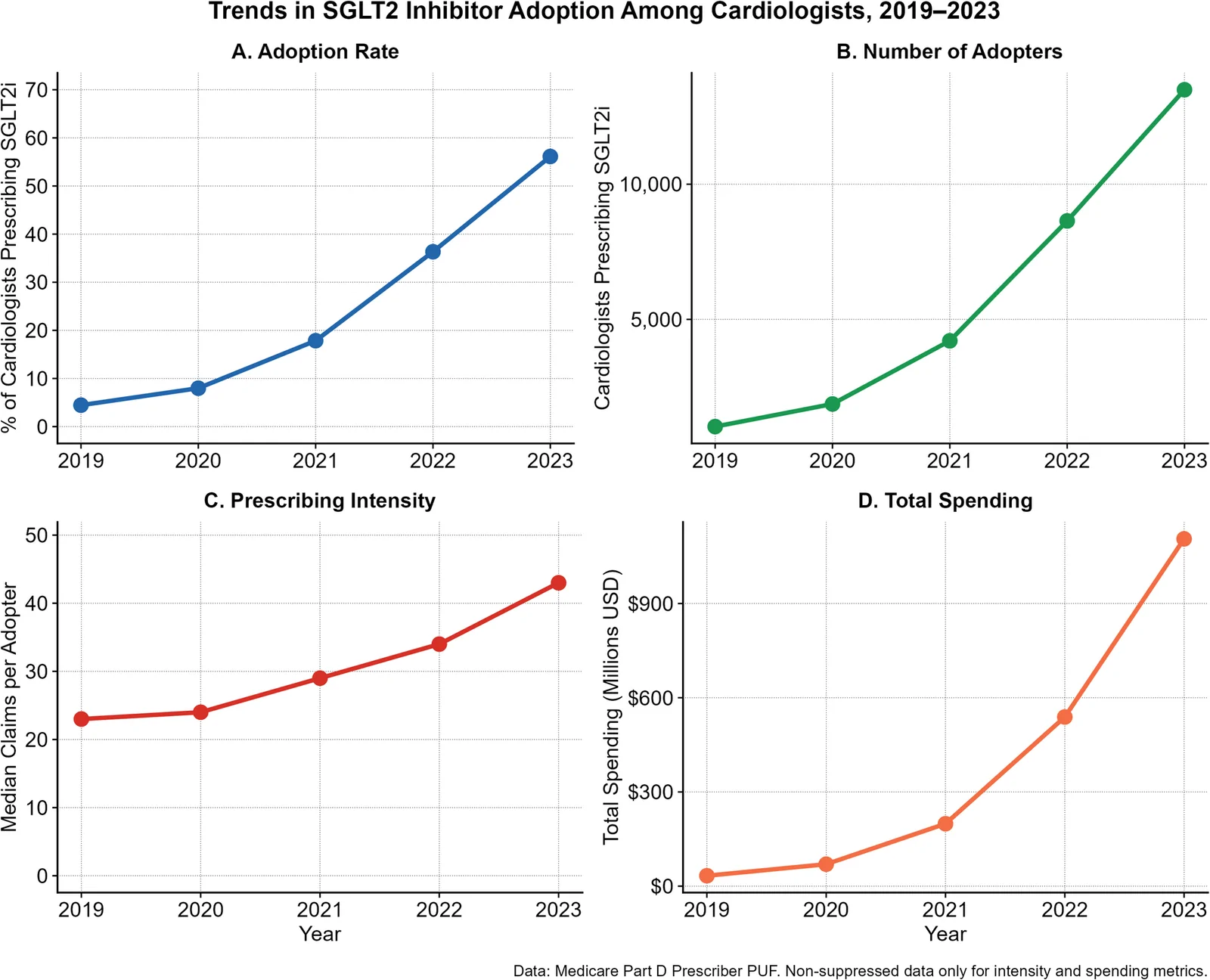
National trends in SGLT2 inhibitor adoption, utilization, and spending among cardiologists, 2019–2023. Panel **A** shows the annual percentage of cardiologists prescribing ≥ 1 SGLT2 inhibitor claim. Panel **B** shows the number of adopting cardiologists over time. Panel **C** displays median prescribing intensity (claims per adopter), and Panel **D** shows total Medicare Part D spending on SGLT2 inhibitors. Data are from the Medicare Part D Prescriber Public Use Files, 2019–2023

Adoption of SGLT2 inhibitors among cardiologists rose more than twelve-fold over the study period, from 4.5% (1,034 of 23,207 cardiologists) in 2019 to 56.1% (13,494 of 24,040) in 2023 (Table 1; Fig. 1A, B). Growth accelerated after 2021, consistent with broader incorporation of SGLT2 inhibitors into heart failure guidelines.

**Table 1:** Adoption and prescribing trends of SGLT2 inhibitors among cardiologists, 2019–2023

Year	Total Cardiologists	Adopters, *n* (%)	Median Claims per Adopter (IQR)	Mean Claims per Adopter	Total Claims	Total Spending ($M)	Mean Spending per Adopter ($)
2019	23,207	1,034 (4.5)	23 (15–43)	41.4	42,772	33.2	32,117
2020	23,434	1,870 (8.0)	24 (14–46)	44.1	82,444	69.8	37,347
2021	23,570	4,207 (17.8)	29 (16–57)	52.0	218,760	198.7	47,237
2022	23,786	8,642 (36.3)	34 (18–72)	63.5	548,900	538.6	62,328
2023	24,040	13,494 (56.1)	43 (21–90)	76.9	1,038,067	1,105.8	81,947

Adoption defined as presence of a non-suppressed SGLT2 inhibitor prescribing record in the Medicare Part D Prescriber PUF (observable adoption). Spending shown in nominal USD; see Table S2 for inflation-adjusted (2019 USD) spending and cost per claim

Prescribing intensity among adopters also increased steadily. Median annual claims per adopting cardiologist rose from 23 claims (IQR, 15–43) in 2019 to 43 claims (IQR, 21–90) in 2023 (Table 1; Fig. 1C). Total annual claims increased from 42,772 in 2019 to more than 1.0 million in 2023.

Medicare Part D spending on SGLT2 inhibitors increased sharply across the period, rising from $33.2 million in 2019 to $1.1 billion in 2023 in nominal dollars, a 33-fold increase; in inflation-adjusted (2019 USD) terms, spending rose 28-fold, to $928 million (Table S2). Cost per claim rose from $776 to $1,065 (+ 37%) over the same period, indicating that rising unit costs, not utilization alone, contributed to spending growth. Mean spending per adopting cardiologist increased from $32,117 in 2019 to $81,947 in 2023 (Table 1; Fig. 1D).

### Geographic variation in adoption

**Fig. 2:**
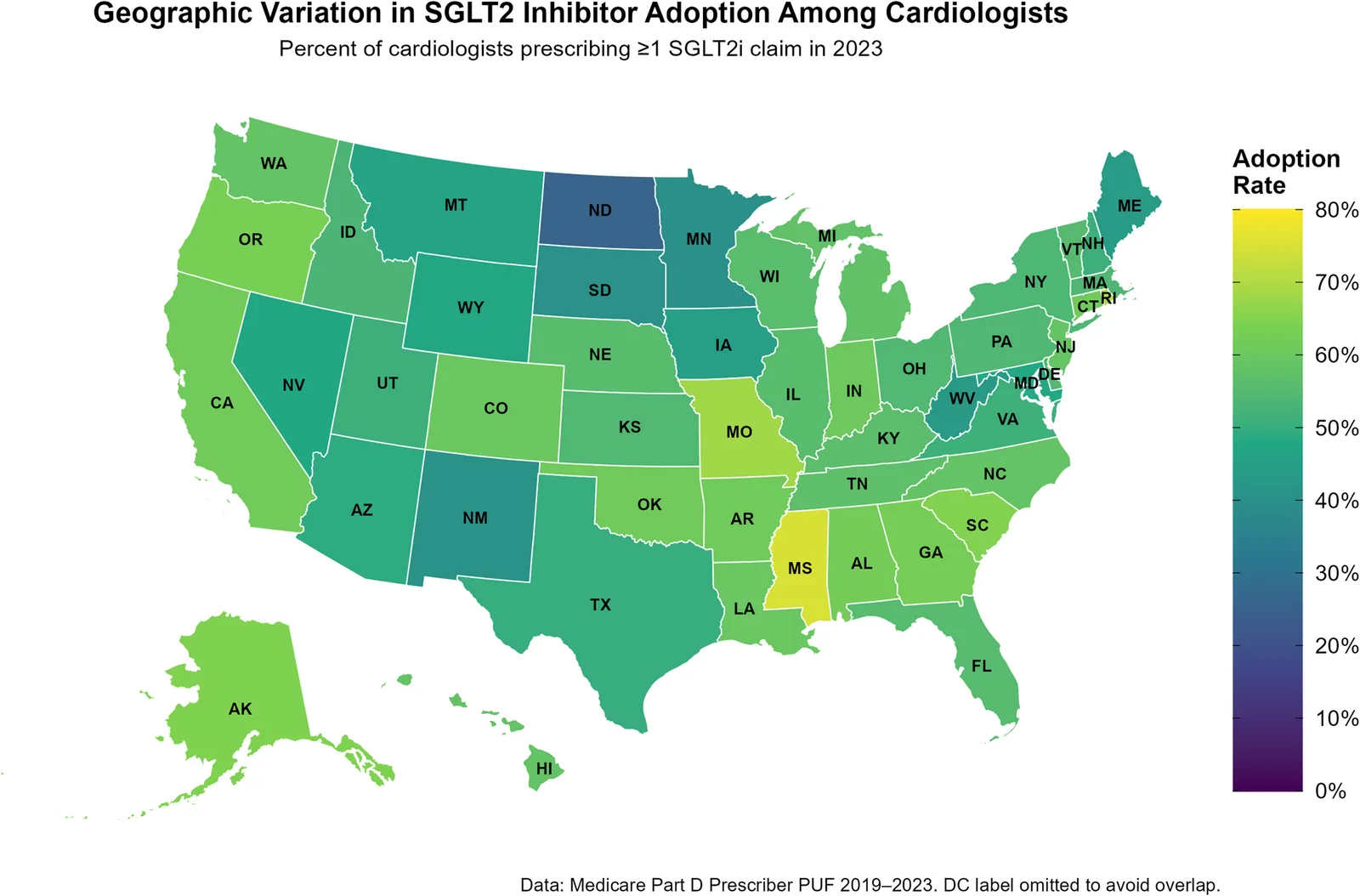
Geographic variation in SGLT2 inhibitor adoption among cardiologists in 2023. State-level observable adoption rates are shown as the percentage of cardiologists with a non-suppressed SGLT2 inhibitor prescribing record in 2023. Estimate stability by state denominator size is assessed separately using Wilson confidence intervals and empirical Bayes shrinkage (Table S3; Figure S1). Data are from the Medicare Part D Prescriber Public Use Files

Adoption varied substantially across states in 2023 (Fig. 2; Table S3). Crude state-level adoption rates ranged from 25.6% in North Dakota to 75.0% in Mississippi. However, states with small cardiologist denominators had wide 95% confidence intervals and were substantially attenuated after empirical Bayes shrinkage: North Dakota’s rate shrank from 25.6% to 43.1%, and similar attenuation was observed for other low-denominator states (Table S3; Figure S1). A chi-square test confirmed state-level adoption differences were statistically significant overall (χ²=316.6, df = 49, *P* < 0.001), but crude state rankings, particularly for states with fewer than 100 cardiologists, should be interpreted cautiously. Despite this state-level variation, adoption was similar when aggregated to the four U.S. Census regions (Northeast 55.3%, Midwest 55.2%, South 55.9%, West 58.1%), suggesting that state-to-state differences do not follow a simple regional pattern.

### Concentration of prescribing among high-volume providers

**Fig. 3:**
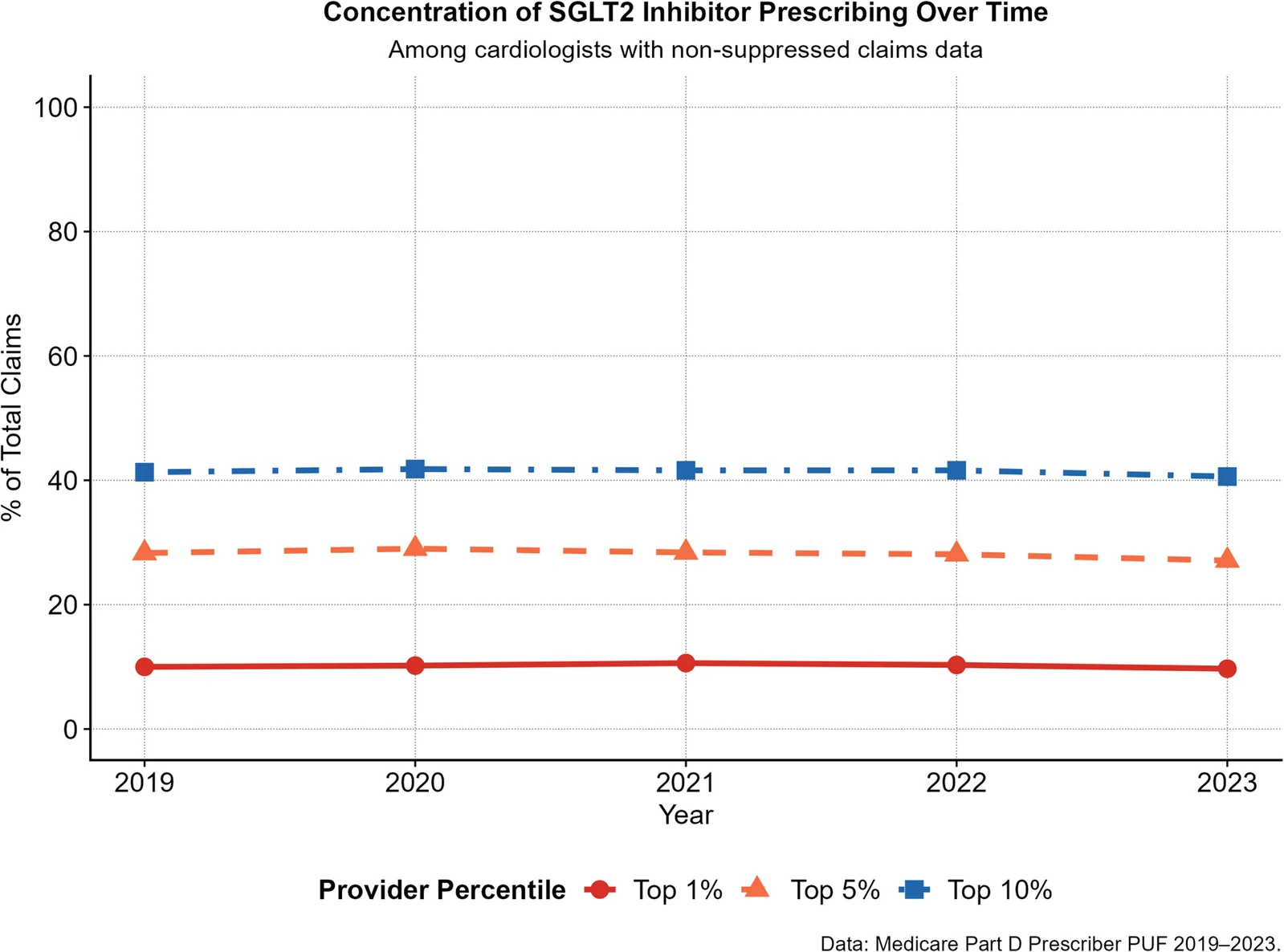
Concentration of SGLT2 inhibitor prescribing among adopting cardiologists, 2019–2023. The proportion of total SGLT2 inhibitor claims accounted for by the top 1%, 5%, and 10% of prescribing cardiologists is shown for each year. Concentration remained stable over time, with the top 10% of prescribers accounting for approximately 40% of all claims annually. Analyses were restricted to cardiologists with non-suppressed claims data

Despite widespread increases in adoption, absolute SGLT2 inhibitor claim volume remained concentrated among a minority of cardiologists throughout the study period (Table 2; Fig. 3). In 2023, the top 10% of prescribers accounted for 40.6% of all SGLT2 inhibitor claims, while the top 5% accounted for 27.1% and the top 1% for 9.7%. This pattern was stable across years, with the top 10% consistently responsible for approximately 41% of annual claims from 2019 to 2023, and the Gini coefficient of claims concentration rose from 0.494 to 0.537 over the same period (Table S2), indicating that inequality broadened across the full distribution even as the top-decile share held steady.

**Table 2:** Concentration of SGLT2 inhibitor prescribing among adopting cardiologists

Year	Total Adopters	Total Claims	Share of Claims by Top 1%	Top 5%	Top 10%	Share of Costs by Top 10%
2019	1,034	42,772	10.0%	28.3%	41.3%	40.5%
2020	1,870	82,444	10.2%	29.0%	41.8%	41.2%
2021	4,207	218,760	10.6%	28.4%	41.6%	41.3%
2022	8,642	548,900	10.3%	28.1%	41.6%	40.8%
2023	13,494	1,038,067	9.7%	27.1%	40.6%	39.5%

Concentration calculated among cardiologists with non-suppressed claims data, ranked by raw annual claim volume. See Table S4 for concentration estimates after normalizing for each cardiologist's overall Part D prescribing volume

However, this concentration was substantially attenuated after normalizing for each cardiologist’s overall Part D prescribing volume: when ranked by SGLT2 inhibitor claims per 1,000 total Part D claims rather than raw claim volume, the top-decile share of claims fell to 21.4% in 2023 (Table S4). Only 31.0% of cardiologists in the top decile of raw SGLT2 inhibitor claims were also in the top decile of overall Part D volume (Spearman ρ = 0.35), indicating that a substantial portion of raw prescribing concentration reflects practice size rather than differential adoption behavior. Cost concentration closely mirrored utilization concentration. In 2023, the top 10% of prescribers accounted for 39.5% of total SGLT2 inhibitor spending (Table 2). Concentration estimates were calculated among adopters with non-suppressed claims data.

In sensitivity analyses excluding low-volume adopters (bottom quartile of annual claims), concentration estimates changed by fewer than 5% points across years (Table S5). Excluding pediatric cardiology and cardiac/thoracic surgery specialties, or restricting the exposure definition to monotherapy SGLT2 inhibitor products, changed 2023 adoption estimates by ≤ 0.3% points; expanding the exposure definition to include canagliflozin and ertugliflozin changed adoption by ≤ 0.1% points in every year (Table S6, Panel B), supporting the robustness of the primary findings to these definitional choices. Restricting the cohort to cardiologists observed in all five study years yielded a similar adoption trajectory (58.7% vs. 56.1% in 2023), indicating that the dynamic annual denominator did not drive the observed trend (Table S7).

### Adoption by cardiology subspecialty

Adoption differed substantially across cardiology subspecialties (Table S8; Figure S2). By 2023, observable adoption reached 91.6% among cardiologists with a subspecialty designation in advanced heart failure/transplant cardiology, compared with 56.4% among general cardiologists and 49.7% among interventional cardiologists (χ²=464.6, df = 2, *P* < 0.001). This divergence emerged early: by 2020, adoption among advanced heart failure/transplant cardiologists had already reached 20.7%, more than double the rate among general cardiologists (8.2%) and interventional cardiologists (4.9%). Prescribing intensity followed the same pattern, with a 2023 median of 105 claims per adopter among advanced heart failure/transplant cardiologists, compared with 42 among general cardiologists and 37 among interventional cardiologists.

### Early versus late adoption and prescribing intensity

In supplemental regression analyses, cardiologists who adopted SGLT2 inhibitors earlier in the diffusion period prescribed at substantially higher intensity than later adopters (median 48 vs. 27 claims, unadjusted; Table S9). Because annual claims are a right-skewed count outcome, we modeled this association using negative binomial regression with standard errors clustered by NPI (*n* = 29,247 clinician-years from 14,293 cardiologists). In an unadjusted model, early adopters (initiating prescribing in 2019–2021) had 2.06-fold higher annual claims volume than late adopters (2022–2023) (IRR, 2.06; 95% CI, 1.98–2.14; *P* < 0.001). After adjustment for calendar year, this association strengthened (IRR, 3.11; 95% CI, 2.99–3.23; *P* < 0.001) and remained similar in models additionally adjusting for specialty category and state (IRR, 2.96; 95% CI, 2.85–3.08, *P* < 0.001).

Together, these findings suggest that early uptake was driven disproportionately by a subset of high-volume prescribers and that diffusion broadened over time without fully eliminating concentration.

## Discussion

This study demonstrates substantial growth in observable SGLT2 inhibitor adoption among cardiologists prescribing to Medicare Part D beneficiaries, with adoption rising from 4.5% in 2019 to 56.1% in 2023. Uptake was accompanied by increasing prescribing intensity among adopters, and early adopters prescribed SGLT2 inhibitors at nearly three times the intensity of late adopters after adjustment (IRR 2.96). Raw prescribing volume remained concentrated, with approximately 41% of claims attributable to the top 10% of prescribers, though this concentration fell to 21.4% after normalization for overall Part D prescribing volume Adoption also differed markedly by subspecialty, reaching 91.6% among advanced heart failure/transplant cardiologists by 2023 compared with roughly half of general and interventional cardiologists. Total nominal spending increased 33-fold during the study period (28-fold in inflation-adjusted terms), and marked geographic variation persisted, with crude 2023 adoption ranging from 75% in Mississippi to 25.6% in North Dakota, though low-denominator state estimates were less stable after empirical Bayes adjustment. These findings suggest that apparent heterogeneity in SGLT2 inhibitor uptake reflects both true differences in adoption behavior and structural factors such as practice volume, subspecialty composition, and denominator instability in low-volume states.

Compared with prior GDMT for heart failure, observable SGLT2 inhibitor adoption in our cohort appears to have occurred more rapidly. Historical data indicate that ACE inhibitor use among patients with congestive heart failure rose from only 24% in 1990 to 39% by 2001 [15]. More recently, adoption of angiotensin receptor-neprilysin inhibitors (ARNI) followed a similarly gradual course: approximately 2.3% of eligible hospitalized patients with HFrEF were prescribed ARNI in the first year after 2015 FDA approval [16], and only 13% of outpatients in the CHAMP-HF registry were receiving it two to three years later [17]. In contrast, observable SGLT2 inhibitor adoption among cardiologists in our cohort reached 56.1% within four years. However, these comparisons should be interpreted cautiously: prior estimates are drawn from patient-level cohorts with confirmed heart failure diagnoses, whereas our measure reflects clinician-level observable prescribing without confirmation of guideline-eligible diagnoses, so the denominators are not directly comparable.

Despite this accelerated uptake, diffusion remained uneven, with early adopters prescribing at substantially higher intensity than late adopters. Early adopters may integrate new therapies rapidly, whereas late adopters may require greater guideline reinforcement or peer influence.

Prescribing also remained concentrated among a subset of high-volume clinicians throughout the study period, and the Gini coefficient of claims concentration rose steadily even as the top-decile share held stable, indicating that inequality broadened across the full distribution rather than remaining confined to the highest-volume prescribers. However, when concentration was recalculated after normalizing for each cardiologist’s overall Part D prescribing volume, the top-decile share fell from 40.6% to 21.4%, and this concentration was substantially explained by subspecialty composition: cardiologists with a subspecialty designation in advanced heart failure/transplant cardiology, a small but disproportionately high-intensity group, reached 91.6% observable adoption and prescribed at more than double the median intensity of general cardiologists by 2023. Together, these findings suggest that raw prescribing concentration reflects a combination of practice volume, subspecialty composition, and true differences in adoption behavior, rather than uneven access alone. This pattern is broadly consistent with established models of evidence diffusion, in which uptake spreads gradually from early, high-intensity adopters to the broader clinician population but may remain incomplete even after strong guideline endorsement [18].

Geographic variation was present but should also be interpreted cautiously. Crude state-level adoption ranged from 25.6% to 75.0% in 2023, but estimates in low-denominator states were unstable and attenuated after empirical Bayes shrinkage. State-level differences remained statistically significant, yet adoption was similar across Census regions, suggesting that uptake does not follow a simple regional gradient. Persistent state-level variation may reflect differences in practice norms, patient populations, health system characteristics, or payer environments; however, these drivers cannot be distinguished in the present analysis.

The incomplete diffusion observed likely reflects barriers operating across multiple domains rather than physician prescribing behavior alone. Systemic barriers include healthcare system organization and workflow, and reimbursement barriers such as medication cost and insurance coverage requirements. Physician-level barriers may include therapeutic inertia, uncertainty regarding prescribing responsibilities across specialties, and concerns regarding initiation and monitoring of therapy [13].

Improving adoption will likely require complementary strategies, including continuing medical education, reduced financial and administrative barriers, multidisciplinary heart failure care models, clinical decision support, and audit-and-feedback interventions. Prior studies support pre-discharge GDMT initiation [19, 20], pharmacist-integrated multidisciplinary care, [21, 22] audit-and-feedback initiatives, [23] and EHR-based alerts [24] as approaches to improving GDMT implementation. Given the lower uptake among general and interventional cardiologists, specialty-targeted decision support and quality improvement efforts may be particularly useful outside advanced heart failure practice. At the policy level, improving medication affordability and reducing administrative barriers may complement clinician- and health system-level implementation strategies. Together, these findings provide a framework for targeting implementation efforts toward clinicians and practice settings where diffusion remains incomplete.

Future research should identify the physician-, patient-, and health system-level barriers that contribute to persistent implementation gaps and evaluate interventions that improve uptake of GDMT.

### Limitations

This study has several limitations. First, because CMS suppresses provider-drug rows with low claim counts, our adoption measure reflects observable prescribing rather than true prescribing status; a cardiologist with sufficiently low-volume SGLT2 inhibitor use could be misclassified as a non-adopter, and the magnitude of this misclassification cannot be quantified from the Public Use Files alone. Second, the analysis is based on Medicare Part D prescribing data, which captures only outpatient Medicare prescriptions and may not reflect prescribing patterns in younger populations or other insurance contexts. Third, the study examines prescribing by cardiologists but does not capture SGLT2 inhibitor prescriptions written by other specialties, potentially underestimating total patient exposure. Fourth, the data do not include clinical information about patient characteristics, indications for prescribing, or appropriateness of use; consequently, observed prescribing may reflect treatment of heart failure, diabetes, chronic kidney disease, or cardiovascular risk reduction, and cannot be interpreted as a direct measure of heart failure GDMT specifically. Fifth, the study period (2019–2023) captures a dynamic period of evolving evidence and guideline updates, and this analysis was not designed to formally attribute changes in adoption to specific trial publications or guideline updates. Sixth, the analysis does not account for potential confounders such as practice setting (academic vs. community) or individual prescriber characteristics beyond subspecialty designation that may influence adoption decisions.

Additionally, the study does not examine patient-level outcomes, discontinuation rates, or persistence with therapy, which are important considerations for understanding real-world effectiveness [25]. Although normalizing for overall Part D prescribing volume and subspecialty composition substantially attenuated raw concentration estimates, the concentration that remained after these adjustments may still reflect either appropriate targeting of high-risk patients or inequitable access, which cannot be distinguished without patient-level data. Finally, the definition of “adoption” as the presence of a non-suppressed SGLT2 inhibitor prescribing record does not capture the intensity or clinical appropriateness of use, and some prescribers classified as adopters may have prescribed these agents infrequently or for limited indications. Geographic variation may be confounded by differences in patient populations, comorbidities, or healthcare infrastructure that were not measured in this analysis, though the similarity of adoption across Census regions argues against a simple regional explanation.

## Conclusion

Observable SGLT2 inhibitor adoption among cardiologists rose sharply from 2019 to 2023, reflecting more rapid translation of clinical trial evidence into practice than has historically been observed for other heart failure therapies. However, a substantial portion of the apparent heterogeneity in adoption timing, prescribing intensity, and geographic uptake is attributable to overall practice volume, subspecialty composition, and denominator instability in low-volume states, rather than adoption behavior alone. Adoption among advanced heart failure/transplant cardiologists in particular approached ceiling levels by 2023, while general and interventional cardiologists lagged substantially behind, suggesting that future implementation efforts may be most productively targeted at broadening adoption outside of heart failure subspecialty practice. 

## Supplementary Information

Supplementary Material 1.

## Data Availability

The datasets analyzed during the current study are publicly available from the Centers for Medicare and Medicaid Services Medicare Part D Prescriber Public Use Files at https://data.cms.gov/provider-summary-by-type-of-service/medicare-part-d-prescribers.

## Abbreviations

SGLT2: Sodium–glucose cotransporter-2
HF: Heart failure
GDMT: Guideline-directed medical therapy
LVEF: Left ventricular ejection fraction
HFrEF: Heart failure with reduced ejection fraction
HFpEF: Heart failure with preserved ejection fraction
IQR: Interquartile range
NPI: National Provider Identifier
PUF: Public Use File
CMS: Centers for Medicare and Medicaid Services
IRR: Incidence rate ratio
CI: Confidence interval
USD: United States dollar
ACE: Angiotensin-converting enzyme
