# Supplementary material for: Adoption and diffusion of SGLT2 inhibitors among cardiologists in medicare part D, 2019–2023: a provider-level observational study
Source: BMC Cardiovasc Disord. 2026 Aug 13;26:1050. doi: 10.1186/s12872-026-06460-x (PMC13749811; doi:10.1186/s12872-026-06460-x)
Supplement: Supplementary file 1 [file 12872_2026_6460_MOESM1_ESM.docx]

**Table S1. Suppression-Related Exclusions Among SGLT2 Inhibitor Adopters, 2019–2023**

| **Year** | **Adopters Analyzed** | **Adopters Excluded (Suppressed)** | **% Excluded** |
| --- | --- | --- | --- |
| 2019 | 1,034 | 0 | 0.0% |
| 2020 | 1,870 | 0 | 0.0% |
| 2021 | 4,207 | 0 | 0.0% |
| 2022 | 8,642 | 0 | 0.0% |
| 2023 | 13,494 | 0 | 0.0% |

**Footnote:** No adopter-years in the primary analysis had suppressed claims data; suppression, where present, affected only non-adopter rows and thus did not bias intensity or concentration estimates among adopters.

**Table S2. Medicare Part D Spending and Prescribing Concentration Inequality, 2019–2023**

*Panel A: Spending, nominal vs. inflation-adjusted*

| **Year** | **Total Claims** | **Cost, Nominal ($M)** | **Cost, Real 2019 USD ($M)** | **Cost per Claim** | **Cost per 30-Day Fill** |
| --- | --- | --- | --- | --- | --- |
| 2019 | 42,772 | 33.2 | 33.2 | $776 | $489 |
| 2020 | 82,444 | 69.8 | 69.0 | $847 | $517 |
| 2021 | 218,760 | 198.7 | 187.5 | $908 | $545 |
| 2022 | 548,900 | 538.6 | 470.6 | $981 | $567 |
| 2023 | 1,038,067 | 1,105.8 | 928.0 | $1,065 | $588 |

*Panel B: Gini coefficient of claims concentration*

| **Year** | **Gini Coefficient** | **n Adopters** |
| --- | --- | --- |
| 2019 | 0.494 | 1,034 |
| 2020 | 0.513 | 1,870 |
| 2021 | 0.521 | 4,207 |
| 2022 | 0.535 | 8,642 |
| 2023 | 0.537 | 13,494 |

**Footnote:** Real costs adjusted using CPI-U annual averages (base year 2019); CPI values should be independently verified against the current BLS release before final submission. Gini coefficient calculated on raw annual claims among non-suppressed adopters; higher values indicate greater inequality across the full distribution.

**Table S3. State-Level Observable Adoption With Wilson 95% Confidence Intervals and Empirical Bayes Shrinkage, 2023**

| **State** | **Cardiologists** | **Adopters** | **Raw %** | **95% CI** | **Empirical Bayes %** |
| --- | --- | --- | --- | --- | --- |
| AK | 31 | 20 | 64.5 | 46.9–78.9 | 58.8 |
| AL | 302 | 188 | 62.3 | 56.7–67.5 | 61.2 |
| AR | 199 | 122 | 61.3 | 54.4–67.8 | 60.0 |
| AZ | 490 | 241 | 49.2 | 44.8–53.6 | 49.8 |
| CA | 2,186 | 1,347 | 61.6 | 59.6–63.6 | 61.5 |
| CO | 299 | 180 | 60.2 | 54.6–65.6 | 59.5 |
| CT | 376 | 231 | 61.4 | 56.4–66.2 | 60.7 |
| DE | 86 | 47 | 54.7 | 44.2–64.7 | 55.0 |
| FL | 1,861 | 1,033 | 55.5 | 53.2–57.8 | 55.5 |
| GA | 706 | 443 | 62.7 | 59.1–66.2 | 62.2 |
| HI | 70 | 41 | 58.6 | 46.9–69.4 | 57.2 |
| IA | 179 | 80 | 44.7 | 37.6–52.0 | 47.2 |
| ID | 75 | 40 | 53.3 | 42.2–64.2 | 54.3 |
| IL | 951 | 532 | 55.9 | 52.8–59.1 | 55.9 |
| IN | 498 | 302 | 60.6 | 56.3–64.8 | 60.1 |
| KS | 186 | 101 | 54.3 | 47.1–61.3 | 54.6 |
| KY | 319 | 180 | 56.4 | 50.9–61.8 | 56.3 |
| LA | 365 | 218 | 59.7 | 54.6–64.6 | 59.2 |
| MA | 701 | 379 | 54.1 | 50.4–57.7 | 54.2 |
| MD | 451 | 217 | 48.1 | 43.5–52.7 | 48.9 |
| ME | 110 | 48 | 43.6 | 34.7–53.0 | 47.6 |
| MI | 778 | 450 | 57.8 | 54.3–61.3 | 57.7 |
| MN | 477 | 191 | 40.0 | 35.7–44.5 | 41.6 |
| MO | 450 | 307 | 68.2 | 63.8–72.4 | 66.8 |
| MS | 156 | 117 | 75.0 | 67.7–81.1 | 69.7 |
| MT | 56 | 26 | 46.4 | 34.0–59.3 | 50.9 |
| NC | 722 | 423 | 58.6 | 55.0–62.1 | 58.4 |
| ND | 39 | 10 | 25.6 | 14.6–41.1 | 43.1 |
| NE | 129 | 72 | 55.8 | 47.2–64.1 | 55.7 |
| NH | 108 | 55 | 50.9 | 41.6–60.2 | 52.5 |
| NJ | 956 | 563 | 58.9 | 55.7–62.0 | 58.7 |
| NM | 91 | 37 | 40.7 | 31.1–50.9 | 46.2 |
| NV | 182 | 87 | 47.8 | 40.7–55.0 | 49.6 |
| NY | 2,071 | 1,114 | 53.8 | 51.6–55.9 | 53.8 |
| OH | 912 | 496 | 54.4 | 51.1–57.6 | 54.5 |
| OK | 239 | 147 | 61.5 | 55.2–67.4 | 60.4 |
| OR | 237 | 150 | 63.3 | 57.0–69.2 | 61.8 |
| PA | 1,278 | 692 | 54.1 | 51.4–56.9 | 54.2 |
| RI | 94 | 66 | 70.2 | 60.3–78.5 | 64.7 |
| SC | 332 | 215 | 64.8 | 59.5–69.7 | 63.4 |
| SD | 47 | 19 | 40.4 | 27.6–54.7 | 48.6 |
| TN | 504 | 287 | 56.9 | 52.6–61.2 | 56.8 |
| TX | 1,803 | 903 | 50.1 | 47.8–52.4 | 50.2 |
| UT | 112 | 57 | 50.9 | 41.8–60.0 | 52.4 |
| VA | 577 | 295 | 51.1 | 47.1–55.2 | 51.5 |
| VT | 29 | 16 | 55.2 | 37.5–71.6 | 55.4 |
| WA | 396 | 232 | 58.6 | 53.7–63.3 | 58.2 |
| WI | 365 | 205 | 56.2 | 51.0–61.2 | 56.1 |
| WV | 104 | 45 | 43.3 | 34.2–52.9 | 47.5 |
| WY | 19 | 9 | 47.4 | 27.3–68.3 | 53.5 |

**Footnote:** District of Columbia omitted due to incomplete reporting. Wilson score 95% confidence intervals shown; empirical Bayes estimates derived from a random-intercept logistic model and represent shrinkage toward the national mean, most pronounced for states with small cardiologist denominators. Given the length of this table (50 states × 5 years = 250 rows), a raw data file containing the complete state-by-year results has been provided as a separate supplementary file for readability and independent verification.

**Table S4. Volume-Normalized Prescribing Intensity and Concentration**

*Panel A: Normalized intensity trend*

| **Year** | **n Adopters** | **Median Claims/1,000 Total Claims** | **Q25** | **Q75** |
| --- | --- | --- | --- | --- |
| 2019 | 1,034 | 5.17 | 2.82 | 10.1 |
| 2020 | 1,870 | 6.48 | 3.30 | 13.7 |
| 2021 | 4,207 | 9.11 | 4.44 | 21.6 |
| 2022 | 8,642 | 12.0 | 5.55 | 27.1 |
| 2023 | 13,494 | 15.9 | 7.47 | 33.4 |

*Panel B: Concentration comparison, 2023*

| **Metric** | **Value** |
| --- | --- |
| Spearman correlation, SGLT2i claims vs. total Part D claims | 0.35 |
| Cardiologists in top decile of SGLT2i claims also in top decile of overall volume | 31.0% |
| Top-decile share of claims, ranked by RAW SGLT2i claims | 40.6% |
| Top-decile share of claims, ranked by NORMALIZED rate (per 1,000 total claims) | 21.4% |
| n adopters analyzed (non-suppressed, 2023) | 13,494 |
| Denominator | Total Part D claims, all drugs, same cardiologist-year |

**Table S5. Sensitivity Analysis of Prescribing Concentration After Excluding Low-Volume Adopters**

| **Year** | **All Adopters** | **Top 75% Prescribers** | **Claims Threshold (25th %ile)** | **Concentration, All (%)** | **Concentration, Top 75% (%)** | **Difference (pp)** |
| --- | --- | --- | --- | --- | --- | --- |
| 2019 | 1,034 | 790 | 15 | 41.3 | 38.6 | −2.8 |
| 2020 | 1,870 | 1,474 | 14 | 41.8 | 39.3 | −2.5 |
| 2021 | 4,207 | 3,237 | 16 | 41.6 | 38.2 | −3.4 |
| 2022 | 8,642 | 6,563 | 18 | 41.6 | 37.7 | −3.9 |
| 2023 | 13,494 | 10,188 | 21 | 40.6 | 36.3 | −4.3 |

**Footnote:** Concentration defined as the share of total claims attributable to the top 10% of prescribers. Low-volume adopters excluded by restricting to cardiologists above the 25th percentile of annual claims.

**Table S6. Drug Exposure Definition Robustness**

*Panel A: Combination-product check*

| **Generic Name** | **Combination Product?** | **n Rows** |
| --- | --- | --- |
| Empagliflozin | No | 23,265 |
| Dapagliflozin Propanediol | No | 16,755 |
| Canagliflozin | No | 342 |
| Empagliflozin/Metformin HCl | Yes | 309 |
| Empagliflozin/Linagliptin | Yes | 103 |
| Dapagliflozin/Metformin HCl | Yes | 81 |
| Canagliflozin/Metformin HCl | Yes | 36 |
| Ertugliflozin Pidolate | No | 17 |
| Dapagliflozin/Saxagliptin HCl | Yes | 2 |
| Ertugliflozin/Metformin | Yes | 1 |

*Panel B: Expanded drug definition sensitivity (adds canagliflozin/ertugliflozin)*

| **Year** | **Total Cardiologists** | **Adopters, Primary Definition** | **% Adopting** | **Adopters, Expanded Definition** | **% Adopting** | **Difference (pp)** |
| --- | --- | --- | --- | --- | --- | --- |
| 2019 | 23,207 | 1,034 | 4.5 | 1,067 | 4.6 | 0.1 |
| 2020 | 23,434 | 1,870 | 8.0 | 1,893 | 8.1 | 0.1 |
| 2021 | 23,570 | 4,207 | 17.8 | 4,219 | 17.9 | 0.1 |
| 2022 | 23,786 | 8,642 | 36.3 | 8,650 | 36.4 | 0.1 |
| 2023 | 24,040 | 13,494 | 56.1 | 13,495 | 56.1 | 0.0 |

**Footnote:** Primary definition includes empagliflozin and dapagliflozin (monotherapy and fixed-dose combinations). Expanded definition additionally includes canagliflozin and ertugliflozin.

**Table S7. Stable Panel Sensitivity Analysis**

| **Year** | **Dynamic Panel: Cardiologists** | **Dynamic Panel: Adopters** | **Dynamic %** | **Stable Panel: Cardiologists** | **Stable Panel: Adopters** | **Stable %** |
| --- | --- | --- | --- | --- | --- | --- |
| 2019 | 23,207 | 1,034 | 4.5 | 19,560 | 940 | 4.8 |
| 2020 | 23,434 | 1,870 | 8.0 | 19,560 | 1,721 | 8.8 |
| 2021 | 23,570 | 4,207 | 17.8 | 19,560 | 3,826 | 19.6 |
| 2022 | 23,786 | 8,642 | 36.3 | 19,560 | 7,609 | 38.9 |
| 2023 | 24,040 | 13,494 | 56.1 | 19,560 | 11,480 | 58.7 |

**Footnote:** Stable panel restricted to 19,560 cardiologists (81.4% of the largest annual denominator) observed in all 5 study years.

**Table S8. Adoption and Prescribing Intensity by Cardiology Subspecialty**

*Panel A: Annual observable adoption*

| **Year** | **Subspecialty** | **Cardiologists** | **Adopters** | **% Adopting** |
| --- | --- | --- | --- | --- |
| 2019 | Advanced HF/Transplant | 546 | 26 | 4.8 |
| 2019 | General Cardiology | 18,960 | 916 | 4.8 |
| 2019 | Interventional | 3,631 | 92 | 2.5 |
| 2020 | Advanced HF/Transplant | 614 | 127 | 20.7 |
| 2020 | General Cardiology | 18,833 | 1,551 | 8.2 |
| 2020 | Interventional | 3,922 | 192 | 4.9 |
| 2021 | Advanced HF/Transplant | 682 | 416 | 61.0 |
| 2021 | General Cardiology | 18,636 | 3,338 | 17.9 |
| 2021 | Interventional | 4,194 | 452 | 10.8 |
| 2022 | Advanced HF/Transplant | 722 | 604 | 83.7 |
| 2022 | General Cardiology | 18,660 | 6,753 | 36.2 |
| 2022 | Interventional | 4,336 | 1,285 | 29.6 |
| 2023 | Advanced HF/Transplant | 762 | 698 | 91.6 |
| 2023 | General Cardiology | 18,713 | 10,560 | 56.4 |
| 2023 | Interventional | 4,495 | 2,236 | 49.7 |

*Panel B: Prescribing intensity among 2023 adopters*

| **Subspecialty** | **n** | **Median Claims** | **Q25** | **Q75** |
| --- | --- | --- | --- | --- |
| Advanced HF/Transplant | 698 | 105 | 47 | 210 |
| General Cardiology | 10,560 | 42 | 21 | 87 |
| Interventional | 2,236 | 37 | 19 | 75 |

*Panel C: Raw specialty strings by year (pre-collapse)*

| **Year** | **Specialty String** | **n Clinicians** |
| --- | --- | --- |
| 2019 | Cardiology | 18,960 |
| 2019 | Interventional Cardiology | 3,631 |
| 2019 | Advanced Heart Failure and Transplant Cardiology | 546 |
| 2019 | Thoracic Surgery (Cardiothoracic Vascular Surgery) | 69 |
| 2019 | Technician, Cardiology | 1 |
| 2020 | Cardiology | 18,833 |
| 2020 | Interventional Cardiology | 3,922 |
| 2020 | Advanced Heart Failure and Transplant Cardiology | 614 |
| 2020 | Thoracic Surgery (Cardiothoracic Vascular Surgery) | 65 |
| 2021 | Cardiology | 18,636 |
| 2021 | Interventional Cardiology | 4,194 |
| 2021 | Advanced Heart Failure and Transplant Cardiology | 682 |
| 2021 | Thoracic Surgery (Cardiothoracic Vascular Surgery) | 57 |
| 2021 | Specialist/Technologist Cardiovascular | 1 |
| 2022 | Cardiology | 18,660 |
| 2022 | Interventional Cardiology | 4,336 |
| 2022 | Advanced Heart Failure and Transplant Cardiology | 722 |
| 2022 | Thoracic Surgery (Cardiothoracic Vascular Surgery) | 68 |
| 2023 | Cardiology | 18,713 |
| 2023 | Interventional Cardiology | 4,495 |
| 2023 | Advanced Heart Failure and Transplant Cardiology | 762 |
| 2023 | Thoracic Surgery (Cardiothoracic Vascular Surgery) | 70 |

**Footnote:** χ²=464.6, df=2, P<0.001 for subspecialty differences in 2023 adoption. Panel C lists every literal specialty string matched by case-insensitive "cardio" pattern matching, prior to collapse into the 3 categories used in Panels A/B; strings with only 1 clinician (Technician, Cardiology; Specialist/Technologist Cardiovascular) were excluded from Panels A/B as non-physician roles.

**Table S9. Corrected Negative Binomial Regression: Early vs. Late Adopters**

*Panel A: Model coefficients*

| **Model** | **IRR** | **95% CI** | **P Value** |
| --- | --- | --- | --- |
| Model 1: Unadjusted | 2.06 | 1.98–2.14 | <0.001 |
| Model 2: Year-adjusted | 3.11 | 2.99–3.23 | <0.001 |
| Model 3: + specialty + state (fully adjusted) | 2.96 | 2.85–3.08 | <0.001 |

*Panel B: Model details*

| **Parameter** | **Value** |
| --- | --- |
| Model family | Negative binomial |
| Link function | Log |
| Standard errors | Clustered by NPI |
| Poisson dispersion ratio (justifies NB over OLS/Poisson) | 87.5 |
| Unique cardiologists | 14,293 |
| Clinician-years analyzed | 29,247 |
| Covariates (Model 3) | Calendar year, specialty category, state |

*Panel C: Unadjusted median claims by group*

| **Group** | **n** | **Median Claims** | **Q25** | **Q75** | **Mean Claims** |
| --- | --- | --- | --- | --- | --- |
| Late (2022–2023) | 14,078 | 27 | 16 | 51 | 42.6 |
| Early (2019–2021) | 15,169 | 48 | 22 | 106 | 87.8 |

**Footnote:** Early adopters defined as cardiologists initiating SGLT2 inhibitor prescribing in 2019-2021; late adopters in 2022-2023. Analysis restricted to adopter-years with non-suppressed claims data.

**Figure S1. Funnel Plot of State-Level SGLT2 Inhibitor Adoption Relative to Cardiologist Denominator, 2023**

State-level observable adoption rates are plotted against the number of cardiologists in each state (log scale), with the national adoption rate (56.1%) shown as a dashed horizontal line. Shaded bands represent 95% and 99.8% control limits under a binomial model; states falling outside the 99.8% limit are labeled and shown in red. States with small denominators (e.g., North Dakota, Wyoming) show the widest control limits, indicating the greatest estimate instability, while large-denominator states (e.g., California, Texas) that fall outside the limits reflect precisely estimated deviations from the national mean rather than instability. Data are from the Medicare Part D Prescriber Public Use Files.

**Figure S2. SGLT2 Inhibitor Adoption by Cardiology Subspecialty, 2019–2023**

Annual observable adoption is shown separately for cardiologists with a subspecialty designation in advanced heart failure/transplant cardiology, general cardiology, and interventional cardiology. A chi-square test confirmed a statistically significant difference in 2023 adoption across subspecialties (χ²=464.6, df=2, P<0.001). Data are from the Medicare Part D Prescriber Public Use Files.
